# Roads to recognition: experiences of migrant women healthcare workers in Norwegian nursing homes

**DOI:** 10.1080/02813432.2026.2636588

**Published:** 2026-03-17

**Authors:** Carmen Theresa Hedlund Quintanilla, Ingrid Jerve Ramsøy, Frode Fadnes Jacobsen, Graziella Van den Bergh

**Affiliations:** a Centre for Care Research West, Western Norway University of Applied Sciences, Bergen, Norway; b Department of Health and Functioning, Western Norway University of Applied Sciences

**Keywords:** Recognition, migrant women, nursing homes, care work, primary healthcare

## Abstract

**Background:**

This article explores how female migrant healthcare workers experience recognition in Norwegian nursing homes. Norway faces a growing shortage of healthcare workers due to an ageing population, and many migrant women enter this predominantly female labour market sector.

**Methods:**

The study employed ethnographic fieldwork guided by a phenomenological approach. Data were collected through interviews with 24 migrant women employed in two municipal nursing homes, alongside participant observation over five weeks during 2023–2024. The participants, from Africa, Asia, Latin America, and non-Scandinavian Europe, had lived in Norway for three to over 20 years. Data were analysed using reflexive thematic analysis.

**Results:**

The first theme, Finding a Way In: Navigating Expectations, explores the challenges these women face in establishing their professional identities. Despite relevant qualifications, many experience diminished professional status, limited recognition, and restricted career development opportunities. Communication barriers and inadequate social integration also influence their daily work experiences. The second theme, Building Trust and Confidence at Work, examines trust-building through task performance and emotional engagement, highlighting that structural barriers and insufficient support hinder their development.

**Conclusions:**

Structural barriers, constrained interpersonal dynamics, and the nature of care work as affective labour often led to unrecognised contributions. Addressing these challenges requires both structural reforms and a cultural shift in how care work and those who perform it are valued.

## Introduction

Recruiting sufficient numbers of qualified professionals in eldercare in Norway remains a significant challenge, particularly in primary healthcare and nursing homes due to demographic changes [1,2]. As a result, many individuals without formal qualifications are employed in the healthcare sector. Approximately 25% of workers in the municipal healthcare sector lack applicable education and are classified as ‘unskilled’ [3,4].

Nursing homes, which fall under municipal responsibility, are a key component of the primary healthcare system in Norway, where the coverage of nursing home spaces is among the highest in OECD countries [5]. Nursing homes provide care for individuals with the greatest need for assistance [6]. Typically, older adults first receive home-based care and then transition to nursing homes as their care needs increase or their functional abilities decline. In these settings, both ‘unskilled’ and skilled healthcare workers provide care to older patients. Although ‘resident’ is the formal term for older adults living in nursing homes, we use the term ‘patient’ in this article because it reflects the terminology consistently used by staff in our empirical material.

Between 2009 and 2017, Norway experienced a significant increase in migrant employment, although this increase did not keep pace with the rapidly rising demand for healthcare services associated with an ageing population [7]. The rise in migrant employment in the healthcare sector is attributed to expanded opportunities in long-term care and targeted recruitment into primary healthcare roles [7,8]. Moreover, migrant women often seek employment in this predominantly female healthcare sector [9,10].

Although migrant women make up a significant part of Norway’s health and care workforce, they are often concentrated in low-paid, care-intensive roles where the labour market conditions are precarious [11]. Migrant healthcare workers in Norwegian nursing homes are predominantly employed as care assistants (*pleieassistenter*) [12]. They provide essential services in a sector facing increasing demands, particularly in care for the elderly and in primary healthcare. Previous studies [13–15] have examined the experiences of care assistants and nurses in long-term care facilities for older adults, highlighting a range of factors influencing their satisfaction and challenges.

Nurses and care assistants report high job satisfaction in their work environments [14]. This sense of fulfilment is closely tied to individual characteristics, such as patience, empathy, respect, trustworthiness, compassion, kindness, and a strong work ethic. These personal attributes enable care assistants to provide quality care and foster positive relationships with older patients [14].

Migrant nurses and care assistants often possess valuable qualities such as support skills, the capacity to effectively address patients’ needs, language proficiency, and an understanding of social and cultural customs [14]. These attributes enhance their ability to adjust to the workplace, allowing them to navigate diverse environments and build rapport with patients and their families. However, it was also found that migrant nurses and care assistants experience feelings of loneliness, overwhelming workloads, low wages, and a lack of recognition in their professional roles [14]. When migrant nurses face barriers in the Norwegian labour market, many settle for positions well below their educational level as regulated health care workers [16].

In the Norwegian system, there are three main forms of formally recognised healthcare workers. Formal recognition is based on official acknowledgement of qualifications and competencies, typically through structured processes such as education, certification, and job titles. *Registered nurses* (RNs) have the most formalised and professionalised roles in the system after completing a three-year bachelor’s degree that includes theoretical and clinical training at a higher education level [17,18]. Internationally educated nurses (IENs) who move to Norway and seek to work as registered nurses must first be authorised as RNs. Migrants who already have a nursing education from countries outside the European Union (EU) and the European Economic Area (EEA) do not receive RN authorisation upon application. Instead, their qualifications are assessed by the Norwegian Directorate of Health, which typically requires them to complete a one-year or two-year part-time bridging programme at a university or university college before they can obtain RN authorisation [19–22]. While enrolled in this programme, many work under temporary or permanent auxiliary nurse (*helsefagarbeider*) authorisation or, if this is pending, as care assistant (*pleieassistent*) [23]. In contrast, nurses educated within the EU/EEA may obtain automatic recognition if their education harmonises with Norwegian requirements [24].

*Auxiliary nurses* (ANs) usually complete two years of upper secondary education followed by two years of practical training, often in institutions such as nursing homes or hospitals [17,25]*. Care assistants* (CAs) are typically defined as ‘unskilled’ workers without formal health education, though some may complete short workplace-based courses [4]. This role is also assumed by students in nursing and medicine as a pertinent means of earning an income while pursuing their studies.

Informal recognition of work competencies, in contrast to formal recognition, is unstructured and occurs in everyday interactions. It includes verbal praise, expressions of gratitude, or social acknowledgement from colleagues, supervisors, patients, or their relatives. Although it is less visible and not formally documented or recognised in terms of salary, informal recognition can still hold significant meaning. It contributes to a positive work environment, increases motivation, and influences how workers perceive their value and respect at work [26].

Despite their contributions, several migrant women experience a lack of social recognition and limited opportunities for career advancement. Their work is often perceived as ‘natural’ caregiving rather than skilled labour, leading to both economic and social undervaluation [8,27,28]. Studies from other countries indicate that migrant care workers in primary healthcare may face misrecognition related to their social status, which can manifest as exclusion or discrimination [29] (p. 72). The status of being a ‘migrant’ is often shaped by institutionalised cultural values that associate it with inferiority, otherness, and difference, rather than equal worth in the context of care work [29] (p. 73). Migrant women face communication and structural barriers within the Norwegian healthcare sector that negatively impact their professional status as they navigate the complex dynamics of obtaining language proficiency and institutional recognition [30].

## Theoretical framework

Care work, defined as a form of affective labour [31] (p. 117), is traditionally associated with women’s work. It is relational and emotionally engaged, facilitating meaningful interactions that are central to the experience of being cared for. According to Lazar [31] (p. 117), caring and affective relationships interact with workers’ agency and resistance in complex ways. Affective labour involves cultivating emotional and relational ties with both patients and employers as part of the labour process [31] (p. 117). Their emotional attachment to patients may weaken their ability to demand better working conditions, and such attachments can obscure exploitative labour relationships. This suggests that migrant women in nursing homes may occupy vulnerable labour market positions and engage in ongoing interpersonal negotiations with both staff and patients. These dynamics can either enable or hinder their ability to carry out their duties effectively, with consequences for both staff and patients [32–35].

The concept of recognition encompasses a range of dimensions, from interpersonal experiences to structural conditions. In this study, the political philosophical use of *recognition* as a concept will aid in understanding how recognition—or the lack thereof—impacts migrant women’s perceptions of their work and their place in Norwegian work life and society [11,36]. For this, we rely on two influential perspectives, those of Axel Honneth [37] and Nancy Fraser [38–40], which offer complementary yet contrasting understandings of recognition. While Honneth [37] emphasises the intersubjective dimension of recognition, arguing that individuals require recognition to develop a positive self-relationship, Fraser [38–40] offers a structural and justice-oriented perspective. She argues that recognition is not primarily a matter of interpersonal validation but a matter of institutionalised patterns of cultural value that determine whose identities are respected or marginalised. Misrecognition, in this view, is a form of institutional subordination that denies individuals or groups the status of full participants in social life [39] (pp. 24–26). Honneth’s [37] theory of recognition offers a valuable framework for understanding the relational dynamics and structural challenges faced by migrant healthcare workers in Norwegian nursing homes. According to Honneth [37], recognition is a fundamental human need, expressed through three spheres: love (emotional support), rights (legal and institutional respect), and solidarity (social esteem). In her justice-oriented perspective, Fraser [40] emphasises that struggles for recognition are deeply connected to social justice, as they reflect disparities in power, status, and material resources. Recognition involves social processes that can either reinforce or challenge inequalities. Struggles for recognition can promote the redistribution of power and foster cooperation across cultural and social divides [38] (pp. 108–109).

In the context of global migration and transcultural interaction, what recognition entails and how it connects to social justice varies according to the norms, values, and structural frameworks that shape how people perceive and relate to one another. Although neither Honneth nor Fraser explicitly distinguishes between formal and informal recognition, this distinction is essential when analysing the workplace experiences of migrant women in nursing homes. Healthcare workers may encounter both forms of recognition, each carrying different implications for their status and inclusion [26,41]. Despite their crucial contributions, migrant women healthcare workers are often employed in lower-status roles, such as care assistants, with limited opportunities for advancement or acknowledgement of their prior qualifications [11,16]. Understanding these challenges underscores the need to enhance working conditions and develop a culture of both institutional and interpersonal recognition. Such recognition is crucial, as it affirms the dignity and value of migrant care workers’ labour and can ultimately improve their experiences and contributions within the healthcare sector.

Drawing on data from workplace observations, informal conversations and interviews, this article aims to deepen our understanding of how migrant women healthcare workers perceive their value in nursing homes, identify the competencies they consider essential for earning colleagues’ trust and respect, and explore the challenges they face in gaining recognition. Thus, our research question is how migrant women healthcare workers in Norwegian nursing homes experience recognition, and what structural and cultural factors influence these experiences?

## Materials and methods

### Research design and approach

This study employed an inductive ethnographic fieldwork approach to explore how the social actions and experiences of migrant women healthcare workers are shaped by the cultural and institutional context of Norwegian nursing homes, utilising multiple qualitative data collection methods [42] (p. 85). A phenomenological perspective guided the study, focusing on the lived experiences and meaning-making processes of migrant women working in nursing homes. This approach emphasises openness and exploration in the field, allowing the researchers to interpret participants’ perspectives and the significance they assign to their experiences [43,44].

### Fieldwork contexts and data collection

Fieldwork was conducted in the primary care sector of a Norwegian municipality. Data were collected in two municipal nursing homes and one adult learning centre offering upper secondary education for ANs. The nursing homes varied in size and structure: one was a small unit with approximately 16 patients across two floors; the other was a larger facility with around 60 patients in four units on a single floor.

The first author carried out fieldwork in both nursing homes over two periods: three weeks in November 2023 and two weeks in February 2024. Observations focused on everyday interactions between migrant women and their colleagues, including informal encounters in corridors, collaborative work tasks, lunch breaks, and staff meetings, particularly those held at the beginning and end of morning shifts at the nursing home. The first author also shadowed several migrant women during their daily routines to gain insight into their work practices and social dynamics. In addition to informal conversations, 21 migrant women employed at the nursing homes were interviewed. Altogether, 10 participants worked at the small nursing home unit and 11 at the larger nursing home facility. These numbers are incidental and correspond to the staff members who were on duty during the fieldwork visits. All individuals who were present were invited, and all consented to participate.

In addition to collecting data at two nursing homes, we also selected as a supplementary site, an adult learning centre where migrant women were enrolled in an AN training programme. The purpose was to gain insights into the experiences of women combining employment with ongoing studies. During a one-week visit, the first author observed classroom interactions and then conducted interviews with three students. These students were simultaneously employed in the healthcare sector, working as CA or unqualified staff in home-based care, nursing homes, or daycare centres for the elderly.

The first author developed an interview guide based on the operationalisation of the research question and reviews of previous research in the field. The interview guide addressed issues such as the women’s previous and current work experiences, the aspects of their work they considered important for acceptance, and whether and how they were included in decision making, social interactions, and professional development, as well as whether they experienced a lack of support, isolation, or limited opportunities. The women were also asked to describe topics such as a typical day at work, their journey to become a healthcare worker, communication and teamwork with colleagues, opportunities for further education, and their approach to be part of their work environment.

Hence, the data were collected through a combination of participant observation, informal conversations and individual interviews. Observations were designed to capture the social and professional interactions of migrant women in their roles as care workers and students. The interviews explored their experiences of healthcare employment, workplace dynamics, and career development possibilities in more detail. The informal conversations and interviews, which were informed by insights from the observations, were conducted in an employee break room, where staff could write reports, take breaks, or hold meetings. Additional conversations also occurred during observations, in hallways, and in common areas designated for nursing home patients. The interviews with the three students took place in a classroom during breaks or while transitioning between buildings to attend different classes.

### Sample

The recruitment of participants was facilitated by a contact person in the municipality, who arranged appointments with managers at the nursing homes and lecturers at the educational institution, ensuring that both staff and students were informed in advance. Purposive sampling was employed, including all individuals present in the workplace during the fieldwork period. A total of 24 migrant women participated in the study. The sample was diverse in terms of cultural background, age, and family situation. Participants came from a range of global regions: six from Asia, six from Africa, three from Latin America, three from Southern Europe, and six from Eastern Europe. Their migration pathways included both refugee status and family reunification.

Formal titles and prior education were confirmed through self-reporting. Two participants were RNs, while nine worked as ANs, some with additional specialisation. Seven participants were employed as CAs, either on temporary contracts or while studying to become ANs through adult education or vocational pathways offered by the municipality. One CA worked as an on-call substitute. In five cases, the participants did not confirm their titles, and their roles were inferred from task descriptions and workplace context. These five participants are therefore assumed to be ANs/CAs.

The study included participants who had lived in Norway for at least three years, were on duty during fieldwork visits and worked as RNs, ANs, or CAs or were enrolled in education programs to become ANs. Workers who did not meet these criteria were excluded. Their ages ranged from 20 to 67 years. Proficiency in Norwegian was not required; interviews were conducted in Norwegian or English, depending on the participant’s preference.

### Data analysis

The collected data were analysed using the six-phase reflexive thematic analysis described by Braun and Clarke [45] grounded in a phenomenological epistemological perspective. This method was chosen to explore how migrant women make sense of their experiences in the context of healthcare work. The analysis was iterative and inductive, with themes constructed through a process of interpretation rather than treated as pre-existing entities within the data. Our analytic approach was reflexive and interpretivist, grounded in the premise that meaning is generated through analysis rather than discovered at a numerically defined endpoint. Consistent with Braun and Clarke’s [46] critique of sample-size rationales based on ‘data saturation’, decisions about participant numbers were made as situated analytic judgements. Decisions about when to cease data collection were responsive to the evolving interpretation of the dataset rather than predetermined numerical thresholds [46].

The analytical process was kept dynamic, according to the steps as outlined by Braun and Clarke [45] (p. 8):

- The first author led the initial analysis. In the first step, all 24 interview transcripts and field notes from five weeks of observation were read and re-read to ensure familiarity with the material. Formulating a research question and the aim of the study were part of the preparation for the next steps.
- In the second step, an open coding was conducted to generate preliminary codes representing the most basic units of meaning. At this stage, a range of codes were created and collated using NVivo software to organise the data.
- In the third step, a systematic review of the transcripts and observation field notes was conducted by the first author. Initial codes were generated and subsequently organised into overarching sub-themes aligned with the research question.
- In the fourth step, emergent subthemes were inductively developed from the coded data. During this phase, Lazar’s theories on affective labour and Honneth’s and Fraser’s theories of recognition were integrated as interpretive frameworks, as patterns identified in the findings aligned with key concepts and perspectives from these theoretical approaches.
- In the fifth step, these initial subthemes were grouped into broader themes related to the study’s theoretical focus. The themes were then reviewed, refined, and, where necessary, combined, split, or discarded. This was an iterative step, and the emerging themes and findings were subsequently discussed among the co-authors to enhance the credibility and trustworthiness of the analysis.
- In the sixth and final step, the themes and sub-themes were further critically evaluated and discussed among all authors. The final overarching themes and their connection with sub-themes are presented in the results section.

**Table 1.** Coding table.

Main themes	Sub-themes	Codes	Descriptions/quotations
Finding a Way In: Navigating Expectations	Proving Yourself Again – From Professionals to Lower Status	Education	*Even though I did consider myself a [registered] nurse, I was not considered to be qualified [in Norway].*
		Nurse knowledge	
	Negotiating Language and Inclusion	Family	*Language challenges can make you feel overwhelmed, even foolish.*
		Language	
Building Trust and Confidence at Work	Care Without Credits	Relations and cooperation	*It is like they [Norwegian colleagues] do not trust us … we migrants are always placed on teams with at least one Norwegian colleague.*
		Community	
	Mobilising Social Relationships	Stereotypes	*Although I have lived in Norway for 24 years, others will always see me as a foreigner, and I feel that I don't quite fit in anywhere.*
		Cultural References	

### Ethics

The study was registered with the Norwegian Agency for Shared Services in Education and Research (SIKT), registration number 437022. All participants were fully informed about the study and provided both verbal and written consent. They were made aware of their right to withdraw from the study at any time without providing a reason, and that withdrawing would not have any negative consequences for their employment. Participants did not receive any benefits from taking part in the study, and none of those who agreed to participate withdrew. Identifying data were anonymised immediately during the transcription of interviews and observations. The names used for participants in the results are pseudonyms to maintain their anonymity. All data were securely stored in accordance with Norwegian data protection regulations and the responsible institution’s data protection procedures. The interview material was anonymised and stored on the responsible institution’s secure research storage platform. Only members of the research team had access to digital files, transcriptions, and field notes.

The study adhered to the ethical principles outlined in both the Declaration of Helsinki [47] and the Guidelines for Research Ethics in the Social Sciences and the Humanities [48], which emphasise respect for individuals, integrity, and social responsibility in research. To ensure respectful and ethical treatment of the participants, the researchers considered their own responsibility in ensuring that all participants understood the terms of their involvement and that their participation was voluntary, with no impact on their employment status. During fieldwork, an attempt was made to build trust through a culturally sensitive approach, recognising and respecting the cultural diversity of all participants.

### Rigour

Methodological rigour was ensured by adhering to established trustworthiness criteria throughout the analytical process, in line with recommendations by Nowell et al. [49]. To ensure the credibility and trustworthiness of the data collection and analysis, it was important to remain open to unexpected findings and be rigorous in the analytical processes [50]. The dynamic process between the different steps in the analysis and the systematic coding helps to ensure that the analysis is comprehensive and captures the complexity of the data. The collective discussions among co-authors for defining and revising the main themes and subthemes helped to mitigate individual biases and enriched the interpretation of results through multiple perspectives. Overall, the rigour of this study is supported through a careful methodological design, systematic analysis, theoretical grounding and collaborative verification processes. Together, these elements contribute to the production of credible, trustworthy, and reliable research findings.

## Results

The analysis resulted in two main themes that characterise migrant women’s processes of recognition. These themes reflect both the recognition they encounter in their work environments and the strategies they employ to navigate challenges and actively pursue recognition.

The first theme, *Finding a Way In: Navigating Expectations*, examines how migrant women experience entering the healthcare sector. It highlights the recognition process involved in transitioning from previous professional identities to lower-status roles, and how language proficiency and social integration influence their inclusion and recognition in the workplace.

The second theme, *Building Trust and Confidence at Work*, captures both the process and the outcome of recognition. It examines how migrant women actively build trust and demonstrate competence in their everyday work interactions, and how these efforts lead to varying degrees of professional validation. Despite limited career advancement opportunities and insecure work environments, the theme illustrates their pursuit of growth and recognition within the workplace.

### Finding a Way In: Navigating expectations

This theme reflects how migrant women working in nursing homes strive to establish themselves as healthcare professionals while navigating the expectations placed upon them. Adaptive strategies and resourcefulness shape their experiences. The women work to meet expectations and challenges, such as navigating complex workplace procedures, dealing with the lack of recognition for prior qualifications and facing limited acknowledgement of their skills. This theme is developed through two subthemes that highlight different aspects of this recognition process, illustrating the complexities and demands migrant women experience when attempting to meet expectations and be recognised at work.

#### Proving yourself again – from professionals to lower status

Some participants had prior experience working in healthcare or held professional qualifications from their countries of origin. However, upon arriving in Norway, they often found themselves employed in positions far below their previous professional status. Structural barriers, such as the non-recognition of foreign credentials and limited access to bridging programmes, presented significant challenges to maintaining their professional identities within the healthcare sector.

Nadiah, for example, was a RN in an Asian country before moving to Norway with her Norwegian husband. Despite her qualifications, she was not recognised as an RN in Norway and was instead employed as an AN. Although she initially enrolled in a Norwegian bachelor’s degree in nursing, she withdrew due to communication barriers, family responsibilities, and the intensity of the programme. She explained:

‘Even though I did consider myself a [registered] nurse, I was not considered to be qualified [in Norway] … I was not allowed to administer medication or handle any reports, even though I knew these tasks well.’

Nadiah’s story illustrates how structural barriers and a lack of formal acknowledgement can undermine professional identity. Her transition to a job as an AN was both a practical solution and a symbolic compromise. In the municipality where the fieldwork was conducted, this was a common path for some of the migrant women. They had to take a new job or go through a new vocational education programme to become an AN, a healthcare position with a lower status than they held as professionals in their home country. Through years of working in primary healthcare and receiving skill training, they could eventually obtain vocational certificates (*fagbrev*) as ANs. However, this required participation in an education and skills training programme that was often slow and fragmented. Some of the women were employed in small part-time positions, which delayed their educational progress and reflected the limited opportunities available to them. These limitations were often shaped concurrently by family obligations and systemic constraints. One such constraint was the lack of accessible opportunities to pursue the bridging nursing programme needed for recognition as an RN in Norway, or the limited acceptance of nursing qualifications that differed from those recognised in Norway.

#### Negotiating language and inclusion

As part of the broader challenge of entering the Norwegian healthcare sector, the women often faced communication barriers that shaped their access to professional recognition. These barriers, including language and cultural barriers, and the invisible labour of adapting to workplace norms, often placed migrant women in marginal positions. Their experiences reveal how inclusion is negotiated daily through language, social interactions, and unspoken expectations embedded in care work.

Several migrant women viewed learning Norwegian as essential for inclusion, yet limited fluency often led to misunderstandings, exclusion, and emotional strain. The pressure to perform linguistically (reading, writing, and speaking) was intensified by part-time or on-call roles, which limited opportunities to practise. Lilja reflected on how communication barriers could lead to conflict and physical stress:

‘Language challenges can make you feel overwhelmed, even foolish. These experiences may lead to [physical pain], such as back pain. That’s why it’s better if they [Norwegian colleagues] check in and offer support, rather than scolding or simply giving orders … it’s about communication.’

Despite these challenges, on-the-job training and practical language programmes, such as *språkpraksis* through the Norwegian Labour and Welfare Administration (NAV), offer valuable opportunities to develop communication skills. The practical language programme is offered to migrants after their language skills have been mapped and assessed. These skills are reportedly the biggest barrier to employment and internships in companies. This programme aims to improve these skills and provide essential work experience in a supportive environment, which in turn will offer greater job opportunities [51]. The practical language programmes might include working in various sectors such as cleaning, care, retail, and office work, a few days a week for a period of three to four months [51]. However, women like Marina highlighted the need for more structured and long-term support:

‘I got the språkpraksis, but no other help from NAV. I want the opportunity to take more courses or pursue further education … I still need to reach the B2 level in Norwegian writing. But this takes time …’

Marina’s reflection highlights the gap between initial integration measures and the long-term support needed for full formal recognition. Her story, like Nadiah’s, underscores how migrant healthcare workers must navigate complex pathways to recognition, often while simultaneously managing underemployment, language demands, and social exclusion. Together, these experiences reveal how the ‘us and them’ divide is reinforced not only by communication barriers, but also by structural barriers that limit access to recognition and advancement. Overcoming this divide requires more than individual effort; it calls for systemic change that values the contributions of migrant women and supports their full participation and inclusion in working life.

Beyond their formal duties, the women engaged in invisible labour to interpret unspoken norms, humour, and social cues. Informal spaces, such as lunch breaks, often appeared as sites of subtle exclusion. A field note from a nursing home illustrates this:

‘During lunch, most employees gather in the hallway, sitting together. A young apprentice student approaches an empty chair, but the cleaner informs her that it is usually reserved for the person in charge of the kitchen. At the far end of the group, two migrant ANs sit quietly, eating their lunch without engaging in conversation—neither with each other nor with the rest of the group. One of them begins scrolling through her phone. Meanwhile, four other employees are actively chatting and laughing … The two migrant women remain entirely on the outside of this interaction and are not acknowledged or invited into the discussion.’ (Field notes, February 2024)

The level of workplace inclusion and recognition was not static but shifted across teams, shifts, and interactions. Participants described feeling as either insiders or outsiders, depending on the support they received from colleagues. The boundaries were not only drawn between Norwegian and migrant workers but also within migrant groups, and were shaped by language, cultural background, and visible differences.

Magdalena, who had worked as a CA in Norway for more than 15 years, shared her experiences concerning the significance of differences:

‘When I think about the differences, we have another personal culture participating in the community—a different economic situation, a different living situation, and what you have experienced earlier in life. This makes us different, and it is a personal culture.’

Magdalena uses the term ‘personal culture’ to describe how individual life experiences among migrants and personal values shape communication and collaboration at work. She emphasises the need for mutual respect and adaptability in diverse teams. When misunderstandings arise, she suggests that sometimes the best response is to withdraw from the situation to avoid conflict.

Magdalena reflected on the challenges of navigating work dynamics:

‘Regardless, when you come to work in Norway, the workplace sets the rules in this building, and you must follow them. I think personal culture may create rudeness among employees. There are misunderstandings, but many times I think it happens because people want it to happen.’

Visible markers of difference, such as skin colour, accents, or religious head coverings, were described as barriers to acceptance and respect by colleagues and patients. These experiences reveal how ethnic and cultural differences, as well as discrimination, can shape workplace dynamics in ways that go unacknowledged. The migrant women expressed concern that such behaviours were often normalised or ignored, reinforcing a sense of exclusion.

Marina reflected on how visible differences affected her colleagues:

‘Some of us feel lucky. I mean, I have light skin, and I think that helps. But I’ve seen how others, especially those with darker skin or who wear a hijab, are treated differently. Patients sometimes say things, and colleagues do not always step in.’

This subtheme reflects how migrant women in the Norwegian healthcare sector navigate communication barriers as they strive to integrate and gain professional recognition. Language proficiency emerged as both a practical necessity and a gatekeeper to trust, responsibility, and inclusion. The women described how limited fluency, cultural misunderstandings, and visible differences contributed to feelings of marginalisation, while informal workplace dynamics and unspoken norms demanded continuous affective labour. These experiences reveal how inclusion is not automatically granted but must be negotiated daily in both formal and informal spaces.

### Building trust and confidence at work

This theme elucidates how migrant women in the Norwegian healthcare sector navigate the complex process of establishing trust and confidence in their roles as healthcare workers. Trust is not only built through task performance or language proficiency, but also through relational practices, emotional engagement, and the dynamics of everyday workplace interactions. The women’s experiences reveal that confidence is shaped not only by their own efforts, but also by how they are perceived, supported, and included in the work environments. The analysis is structured around two interrelated subthemes that shed light on how migrant care staff handle trust, competence and acknowledgement in their daily work.

#### Care without credits

This subtheme depicts how migrant women demonstrate their professional competence not only through language but also through caregiving practices, emotional engagement, and embodied knowledge. The migrant women emphasised that being a good care worker involves more than verbal fluency; it also requires attentiveness, empathy and the ability to connect with patients through small, meaningful actions. These expressions of care are central to how migrant women build a sense of competence and trust and seek to be acknowledged as capable healthcare workers in the workplace, despite the undervaluation of this type of work.

Nadiah argued that language should not be the only criterion for competence. For her, competence in caregiving involves forming human connections and showing empathy through everyday care:

‘When I leave home, I have pretty clothes… [One of the patients] is 90 years old. She could die anytime and anywhere. So, while she is alive, she should wear pretty clothes. I think we should take the time so everyone should be clean and wear pretty clothes.’

Several research participants emphasised the importance of nonverbal communication competence, like touching, eye contact, and smiling, as central to their caregiving. These embodied practices were often overlooked by leaders and Norwegian colleagues in favour of verbal fluency, leaving some research participants feeling like their contributions were undervalued.

The migrant women frequently experienced being relied upon and even trusted in performing practical tasks, but this trust did not always translate into formal recognition or respect at the nursing home. Instead, their competence was often taken for granted, and their actions were subject to subtle forms of mistrust.

Such dynamics can lead to feelings of invisibility and exclusion, particularly in structural settings where verbal fluency and formal credentials are prioritised. Several women described how their questions or contributions were ignored, reinforcing a sense of marginalisation. A field note from a course on dementia held at one of the nursing homes illustrates this:

‘One day, several ANs attended an online course on dementia, led by the professional development coordinator. The session was rushed, and the professional development coordinator (an RN) appeared disengaged, likely because she had already delivered the same course to another group earlier that day. Several migrant workers were present and seemed to be carefully reading, listening, and trying to understand the scenarios presented. Three of the migrant workers were engaging actively by answering questions and providing comments. However, when one of the women sitting at the front asked about a Norwegian word she did not understand, no one responded. The professional development coordinator continued clicking through the material without acknowledging the question. The woman quietly lowered her hand and remained silent for the rest of the session.’ (Field notes, November 2023)

This education session took place during working hours and could be considered compulsory and necessary for learning to practice care work in a nursing home. Despite the migrant workers’ visible engagement and commitment to learning, their efforts were left unacknowledged. The silence that followed the woman’s question illustrates how trust and competence can be quietly undermined in everyday interactions. Her attempt to participate was not only ignored but subtly discouraged, reinforcing a sense of exclusion.

Such moments were not isolated. They reflect broader patterns in which migrant women are trusted to perform care tasks but not always treated as knowledgeable or competent healthcare workers. Their contributions often go uncredited, even as they continue to uphold good standards of care. These everyday interactions are characterised by the quiet, persistent ways migrant women sustain relationships and maintain the quality of their work, even in environments where their efforts are met with indifference or suspicion.

This gap between being trusted to carry out care and being ignored as a competent healthcare worker can erode confidence and create social distance in the workplace. Several research participants described feeling undervalued or overlooked by colleagues and employers. This sense of being disregarded was often accompanied by a feeling of being under surveillance.

Several migrant women reported being paired with Norwegian colleagues in ways that suggested a lack of trust. As one woman, Marina, explained:

‘It is like they [Norwegian colleagues] do not trust us … we migrants are always placed on teams with at least one Norwegian colleague.’

Such experiences contributed to feelings of insecurity and reinforced the perception that migrant women must work hard to prove their competence. They also illustrate how migrant women build trust and demonstrate competence through everyday acts of care that are often undervalued or overlooked. This subtheme highlights the ongoing struggle that migrant women face in gaining trust and acknowledgement in the workplace.

#### *Mobili*s*ing social relationships*

This subtheme highlights barriers migrant workers encounter, such as employment restrictions and limited career advancement, and the resilience and ingenuity with which they respond to these barriers. By mobilising social relationships within and beyond the workplace, they adapt to these constraints and create opportunities for participation and acknowledgement in a system that often overlooks their contributions.

Mobilisation through social relations took place in informal learning spaces, which served as crucial entry points into the care sector for some migrant women. Amina, who arrived in Norway from an African country without any formal education, described how attending a local language café (*språkkafé*) helped her begin learning Norwegian and build social connections. Organised by libraries and volunteer organisations, these cafés provide migrants with a space to practise language skills in informal and supportive settings [52]. Such initiatives are widespread in Scandinavian and other European countries. They do not offer any formal instruction or belong to any institutional programme [53] but, rather, aim to generate conversations that are understandable for both migrants and native speakers, for example, about current events or topics [54]. Amina’s informal language learning and socialisation eventually led her to pursue formal education and secure a job at a daycare centre for the elderly. Reflecting on her learning and career experience from one context to another, she said:

‘We don’t have nursing homes in my home country … But here, people are busy, they work all the time and don’t have time to care for their parents. Therefore, it’s beneficial to have a space where older individuals can connect with others and meet new people.’

As a single mother, Amina had to balance her work schedule with childcare responsibilities. Despite these challenges, she found meaning in her role and appreciated the opportunity to contribute to a care system that differed from her cultural background. Her experience illustrates how personal growth, cultural adaptation, and informal learning come together in the process of becoming a member of the care workforce.

Support from family, teachers, and colleagues played an essential role in sustaining motivation to work in the healthcare sector. Several women described how encouragement through social relationships helped them feel seen and capable, reinforcing their belief in their own work abilities. Some migrant women employed in the healthcare sector encountered barriers to securing stable and meaningful employment opportunities. Olivia, originally from a Latin American country, had lived in Norway for 24 years. She shared how her working life had shifted over time. Despite her long-term residence in Norway, her employment situation remained unpredictable:

‘I have a degree in [higher education] and worked as an [job position related to her degree] here in Norway until the COVID-19 pandemic. When all the offices were closed, I lost my job. That’s when I started working at the nursing home. Besides working at the nursing home, I also work as an ‘on-call substitute’ in a [institution in the education sector]. I have tried to work a full day shift at [her other job] and then go straight to an afternoon shift at the nursing home. But I found this routine too overwhelming and exhausting.’

She linked her experience to her migrant status:

‘Although I have lived in Norway for 24 years, others will always see me as a foreigner, and I feel that I don’t quite fit in anywhere.’

Olivia’s experience highlights how even long-term migrants with diverse formal qualifications can face unstable employment and limited career progression when working in nursing homes. For some migrant women, financial necessity and limited opportunities lead to accepting unpredictable work schedules, often at the expense of their well-being and long-term employment prospects.

This subtheme reveals that building confidence and developing competence in the healthcare sector is not solely a matter of individual effort. Although migrant healthcare workers are often adaptable, eager to learn, and resilient, they face significant employment barriers and have limited opportunities for career advancement. Informal learning spaces and supportive networks, typically found outside the workplace, can offer motivation and a sense of mobilisation for inclusion. However, these supports are often insufficient to compensate for insecure employment and a lack of recognition within formal work settings. The experiences of Amina and Olivia illustrate how migrant women must often carve out their own paths to competence by mobilising social relationships, frequently despite, rather than with the support of, the organisational structures in which they work.

## Discussion

The experiences of the 24 migrant healthcare workers in this study offer a valuable lens through which to explore how recognition is negotiated in everyday life in nursing homes, illustrating that recognition is not a fixed or uniformly granted condition but emerges through continuous and often contested social processes. Recognition is shaped not only by formal qualifications or job performance but also by how the migrant women healthcare workers are perceived and included in the social and relational dynamics of the workplace. The women’s accounts show that recognition at work involves an ongoing negotiation between their own efforts and the conditions they face. On the one hand, they invest time and energy into learning, adapting, and proving their competence. On the other hand, they encounter external challenges that limit their progress. These challenges include insecure and often short-term employment situations with few opportunities for advancement.

This discussion draws on Honneth’s [37] and Fraser’s [38–40] theories of recognition and redistribution, along with Lazar’s [31] perspective on affective labour, to examine how the migrant women navigate the layered demands of care, cultural adaptation, and the pursuit of work status validation

Honneth [37] identifies three spheres of recognition—love, rights, and solidarity—as essential to individual self-realisation. In this study, the delayed or denied formal recognition of prior education reflects a breakdown in the formal recognition system, where the organisations they work for fail to acknowledge migrant women’s qualifications. Consequently, work status validation is often negotiated informally through workplace relationships, affective labour, and demonstrations of competence. These practices are more closely related to Honneth’s spheres of love and solidarity, where interpersonal bonds and social esteem serve as crucial mechanisms of recognition. The reliance on informal validation underscores a broader struggle for acknowledgement, as migrant women seek to assert their value and dignity in environments that do not formally recognise their contributions. Solidarity, in Honneth’s terms, involves being valued for one’s unique abilities and efforts. When colleagues and employers recognise these informal contributions, they participate in a form of solidarity that can partially compensate for organisational misrecognition. Yet, this recognition process is far from secure; without formal validation, migrant women remain highly vulnerable to social and economic exclusion. This highlights the necessity for recognition practices that are both more inclusive and equitable.

Despite contributions, some of the women experienced a lack of social recognition and limited opportunities for career advancement. Care work is often perceived as ‘natural’ caregiving rather than skilled labour, leading to both economic and social undervaluation [8,27,28]. This perception reinforces gendered and ethnic assumptions about care work and contributes to the invisibility of their skills. As Näre [29] argues, the status of being a ‘migrant’ is often shaped by institutionalised cultural values that associate it with inferiority, otherness, and difference, rather than equal worth in the context of care work.

While migrant women did not report experiencing significant differences in recognition based on cultural background or age, they frequently referred to experiences of other migrant women who had encountered such barriers. These experiences reflect a form of status subordination [38], in which social difference becomes a basis for misrecognition, irrespective of competence or commitment.

These differences can act as a barrier to being acknowledged as equals. This was particularly evident among the migrant women with nursing qualifications who had not been recognised as RNs in Norway. This finding aligns with existing research [55–58] that underscores the challenges associated with obtaining formal recognition of nursing qualifications acquired abroad. The limited opportunities for formal recognition of their previous education and the requirement to start anew to obtain a bachelor’s degree in nursing can be time-consuming and may negatively impact their motivation [59]. Furthermore, it is possible that the migrant women were unaware of alternative pathways to obtain authorisation, such as bridging nursing programmes.

Navigating expectations was a concern for migrant women with visible markers of difference, for instance skin colour or head coverings. These characteristics shaped how colleagues, patients, and their families interacted with the women, often reinforcing boundaries that influenced social interactions and acceptance. Several of the migrant women observed that colleagues with more visibly marked identities faced greater challenges in being recognised as equals by both patients and colleagues. These accounts highlight the persistence of discriminatory attitudes that can undermine efforts to be perceived as full members of a care team. Moreover, many expressed concern that such exclusionary behaviours often went unnoticed or unaddressed, highlighting the need for greater awareness of how everyday actions and assumptions can contribute to marginalisation.

Communication barriers also emerged as a key factor in the recognition process. Even when the research participants were fully capable of performing tasks, difficulties in expressing themselves or understanding others created communication barriers. As Zainal et al. [60] argue, clear communication and defined expectations are essential for enabling migrant workers to understand their roles and advocate for their needs. Migrant women face significant communication and structural barriers that undermine their professional status, as they must navigate a complex landscape of language proficiency and institutional recognition [30]. In this context, language becomes not only a tool for care but a gatekeeper to trust, responsibility, and inclusion

Fraser’s [38] critique of the ‘recognition turn’ in social justice theory is particularly relevant in this study. She argues that struggles for recognition must not be separated from struggles for redistribution, as doing so risks obscuring the material conditions that underpin social inequalities. For migrant healthcare workers, this means that calls for cultural inclusion or respect must be understood alongside persistent structural barriers such as insecure working conditions, limited career mobility, and unequal access to formal competence development. In Fraser’s [38] terms, recognition is not merely about affirming identity but about transforming structural arrangements that deny individuals full participation in social and professional life. Recognition was described as essential to feeling valued and included. In environments where communication was strained or where the migrant women felt ignored, silence and withdrawal often became resilience strategies. Field observations illustrated how these behaviours may reflect broader dynamics of power and status in the workplace, where trust and credibility must be earned through extra effort.

Building trust and confidence, then, is not simply a matter of individual resilience; it involves developing strategies for handling challenging situations and asserting one’s place in the workplace. As Crawshaw et al. [61] suggest, trust is shaped by lived experience and is central to workplace recognition. For migrant healthcare workers, gaining trust is not only a matter of demonstrating competence but also about navigating implicit expectations and social hierarchies in their new environment.

This need to continually negotiate trust and recognition becomes pronounced for migrant women, whose experiences reveal how individual efforts to gain esteem intersect with broader structural barriers. Migrant women pursue social esteem in their work environments, where structural recognition is lacking, Fraser [38] adds another perspective to the structural dimension of this lack of recognition. Migrant women not only have to constantly prove their competence and navigate workplace hierarchies, but they may also experience unequal working conditions as a result.

The women described affective labour in caregiving as central to relational strategies within informal recognition processes. Despite its emotional and relational significance, affective labour is often undervalued. As Lazar [31] notes, affective labour is key to how care workers define their work identity. This labour is not limited to patient care; it also shapes relationships with colleagues. The ongoing effort to adapt to workplace norms and fit in socially is a form of affective labour.

The strategies that migrant women use to overcome barriers involve affective labour in two ways: first, through the care they provide to patients, which deserves formal recognition; and second, through relational work with colleagues, as they navigate workplace dynamics to gain recognition, whether formal or informal. For some of the women, recognition of this relational labour was considered an important complement to formal qualifications and job titles, and in some cases, is perceived as equally significant. However, this affective investment can also create structural constraints. Strong emotional ties to patients and a desire to improve their conditions may discourage collective bargaining or demands for stable employment. Instead, recognition is often pursued through interpersonal negotiation with employers, rather than through formal agreements or union representation.

While some migrant women described feeling a sense of dignity when the recognition process went well, others spoke of loneliness, exhaustion, and constant pressure to prove themselves. They expressed a clear need to be valued, included, and recognised within the workplace community. For some, the opportunity to work in Norway and contribute meaningfully to elderly care was a source of pride. However, experiences of misrecognition and distrust from colleagues could undermine this sense of value.

Other studies [62,63] underscore that misrecognition constrains the capacity of marginalised groups to engage in purposeful, goal-directed action. Fraser [38] extends this understanding by conceptualising misrecognition not simply as an identity or status issue, but as a manifestation of structural subordination [62–64]. This dynamic is evident in this study’s findings, where migrant women navigate a dual struggle: resisting misrecognition while simultaneously challenging the structural subordination that shapes their opportunities for recognition. In their efforts to be recognised as competent health workers within municipal health services, they must confront both dimensions of injustice. Crucially Fraser [38] argues that formal equality alone does not secure recognition; rather, genuine recognition requires acknowledging difference as a foundation for agency and social participation [38,62,63].

These findings underscore the importance of collegial support in shaping experiences of inclusion and recognition. Migrant women’s sense of being valued often depended on the quality of their relationships with colleagues, indicating that supportive workplace cultures are essential for fostering informal forms of recognition. The workplace dynamics highlighted in this study further illustrate how migrant women may occupy shifting positions as both ‘insiders’ and ‘outsiders’, depending on the specific social and professional contexts within care teams. Moreover, differences within migrant groups, including language background, ethnicity, and length of residence in Norway, also influenced these relational dynamics and contributed to variations in how recognition was negotiated and experienced.

## Conclusions

To conclude, this discussion suggests that the main themes *Finding a Way In: Navigating Expectations* and *Building Trust and Confidence at Work* are intertwined processes for migrant women in Norwegian nursing homes, shaped by structural barriers and interpersonal dynamics that influence both access to employment and experiences of recognition. Drawing on Honneth’s [37] and Fraser’s [38] theories of recognition, as well as Lazar’s [31] perspective on affective labour, the analysis highlights how migrant women must navigate visible differences, communication barriers, and informal workplace norms in their pursuit of recognition. While several of the women demonstrate resilience and commitment, their contributions are often undervalued, and their skills are misrecognised as ‘natural’ caregiving rather than professional competence. The strain of being relied upon yet not fully acknowledged for their skills and commitment highlights the need for a broader understanding of competence in healthcare work, one that values the quiet, relational work at the core of caregiving. Addressing these challenges requires not only structural reforms but also a cultural shift in how care work, and the diverse workforce that performs it is acknowledged and valued.

### Strengths and limitations of the study

In this study, we consider the depth of understanding that the qualitative research approach provides, and the richness of the experiences shared by migrant healthcare workers in Norwegian nursing homes as a significant strength. By collecting data through observations, informal conversations and semi-structured interviews, we gathered rich, nuanced descriptions of the participants’ experiences. The interviews demonstrated significant information power, and the research utilised well-established theoretical frameworks, providing a solid foundation for the analysis.

Furthermore, the quality of the interaction between the interviewer and interviewees played a crucial role in eliciting meaningful insights. The researchers prior knowledge and expertise in the field enabled a deep and reliable interpretation of the data collected. Another notable strength of this study is the multidisciplinary composition of the research team, which encompasses expertise in both social sciences and health sciences. These diverse academic backgrounds contributed to a more nuanced analytical process, leading to a more comprehensive understanding of the study’s findings.

Despite these strengths, it is important to acknowledge several limitations may impact the findings. The time frame allocated for data collection posed constraints on the depth of exploration concerning certain themes. Limited time may have prevented researchers to engage in thorough discussions with participants, potentially leading to an incomplete understanding of some topics. Consequently, while the study provides valuable insights, these limitations should be considered when interpreting the findings and their implications for broader populations of migrant healthcare workers. Furthermore, the researchers remain acutely aware of the potential for bias in their analysis. Given their backgrounds and experiences, they recognise that their perspectives may inadvertently influence the interpretation of the data. This emphasis on critical reflection in qualitative research highlights the need to mitigate the potential impact of biases.

### Clinical implications

The findings of this study have important implications for policy and practice in primary care and municipal healthcare, particularly within the context of nursing homes. They address the need for a more inclusive and equitable approach to recognising and supporting migrant healthcare workers, whose contributions are essential to the functioning of long-term care services in Norway.

This discussion points to several key implications for municipal healthcare services. Formal recognition pathways for migrant healthcare workers need strengthening, as delays or denials in validating prior education limit career progression and reinforce marginalisation. Transparent, timely credentialing processes, supported by bridging programmes that integrate language and professional training, are essential.

Workplace discrimination, both informal and structural, must also be addressed. Visible differences, such as skin tone or religious head coverings, were reported as barriers to equal recognition, reflecting deeper cultural biases. Employers should establish clear procedures for reporting and responding to exclusionary behaviour.

Affective labour—the emotional and relational work central to caregiving, remains undervalued. Migrant workers often carry out this labour with deep commitment, yet it is rarely acknowledged in formal assessments. Policies should recognise affective labour as a core aspect of care work and incorporate it into performance reviews and professional development frameworks.

Language proficiency is another key factor. Even when tasks are performed competently, communication barriers can limit participation and trust. Therefore, workplace-integrated language support tailored to care settings is needed.

Career development opportunities are often lacking. Despite their skills and motivation, migrant women are frequently confined to low-status roles, part-time or temporary employment. Inclusive mentoring and clear career pathways are essential to support advancement.

Finally, collegial support plays a crucial role. Recognition is often negotiated informally through everyday interactions, making a supportive team culture vital. Employers should invest in team-building and reflective practices that foster inclusion and mutual respect.

In summary, improving recognition and inclusion for migrant healthcare workers requires both structural reform and cultural change. Doing so will enhance staff well-being and retention, while strengthening the quality and sustainability of care for older patients.

## Data Availability

The data in this study are available from the corresponding author on reasonable request, due to Western Norway University of Applied Sciences PhD program policy.
